# Availability of Naloxone at Rural Georgia Pharmacies, 2019

**DOI:** 10.1001/jamanetworkopen.2019.21227

**Published:** 2020-02-14

**Authors:** Jennifer L. Nguyen, Lauren R. Gilbert, Lauren Beasley, John J. Brooks, Jennifer Elliott, K. Bryant Smalley, Jacob C. Warren

**Affiliations:** 1 Mercer University College of Pharmacy, Atlanta, Georgia; 2 Mercer University School of Medicine, Macon, Georgia

## Abstract

This cross-sectional study evaluates the availability of naloxone, pharmacists’ awareness of the standing order regarding dispensing naloxone without a prescription, and the cost of the drug from chain and independent pharmacies in rural counties in Georgia.

## Introduction

As a response to the ongoing opioid crisis, every US state has increased access to naloxone through a variety of expanded prescribing methods, such as standing orders or protocols.^1^ In Georgia, a standing order decrees that any individual may obtain naloxone from a licensed pharmacy without a prescription.^2^ Prior research^3,4^ showed slow adoption of having naloxone in stock and dispensing the medication under the standing order. This cross-sectional study evaluated the availability of naloxone, pharmacists’ awareness of the standing order, and the cost of the drug from chain and independent pharmacies in legislatively designated rural counties in Georgia 52 months after the standing order was signed into law.

## Methods

This study was reviewed by Mercer University’s Institutional Review Board for Human Subjects Research and was deemed exempt from the need for informed consent because no patient data were used.

A list of licensed pharmacies was obtained from the Georgia Board of Pharmacy. Using zip codes, researchers verified the list for accuracy. Inclusion criteria were retail pharmacies located in a rural county (defined by the State of Georgia as having a population <35 000 or legislatively designated as such).^5^ From May 2019 to August 2019, trained researchers acted as secret shoppers (ie, posed as patients), asking to speak with the pharmacist. The researchers followed a scripted interview guide and entered responses in a data collection instrument (Table). For uniform data collection, researchers discussed how to answer questions and record responses with the lead authors to resolve issues in real time. The use of the brand name Narcan vs the generic name naloxone was chosen, because the public is more likely to recognize the brand name of the drug.

**Table.** Telephone Responses to Questions About Accessibility of Narcan (Naloxone) Under the Standing Order by Pharmacists in Rural Pharmacies in Georgia

Questions and Answers	Responses, No. (%) [95% CI]
“Do you guys have Narcan?” (n = 364)	199 (54.7) [49.4-59.9]
If Narcan was not available: “Do you know where else I could find it?” (n = 164)	
Chain store	123 (75.0) [67.7-81.4]
Other specific store	19 (11.6) [7.1-17.5]
Unaware of another pharmacy	15 (9.1) [5.2-14.6]
Vague answer	7 (4.3) [1.7-8.6]
If Narcan was available and a price was given: “Is there a cheaper or different option?” (n = 198)	
Generic naloxone option	33 (16.7) [11.8-22.6]
No cheaper option given	127 (64.1) [57.0-70.8]
Call around to other pharmacies	6 (3.0) [1.1-6.5]
Coupon suggested	20 (10.1) [6.3-15.2]
Did not know of any cheaper option	12 (6.1) [3.2-10.4]
“Do I need a prescription for that?” (n = 259)	
Yes	123 (47.5) [41.3-53.8]
No	122 (47.1) [40.9-53.4]
Sometimes	6 (2.3) [0.9-5.0]
Unsure	8 (3.1) [1.3-6.0]

Data analysis was conducted using Excel spreadsheet software version 2016 (Microsoft Corp) and SPSS statistical software version 25 (IBM Corp). Descriptive statistics are presented. Data analysis was performed October 2019 to December 2019.

## Results

All 374 pharmacies in Georgia’s 109 rural counties were called, with a 97.3% response rate (364 pharmacies) (Figure). One hundred ninety-nine of the 364 pharmacies (54.7%; 95% CI, 49.4%-59.9%) claimed to have the medication in stock. Pharmacists who stated they did not have the product were asked whether they could refer elsewhere to obtain the product. Of 164 pharmacists who responded, 123 (75.0%; 95% CI, 67.7%-81.4%) referred the caller to a chain store (either by name or using a generic term), 19 (11.6%; 95% CI, 7.1%-17.5%) referred callers to another specific retail store, 15 (9.1%; 95% CI, 5.2%-14.6%) were unaware of another pharmacy that carried naloxone, and 7 (4.3%; 95% CI, 1.7%-8.6%) gave vague responses, such as recommending another location or advising the caller to “call around.”

**Figure.**
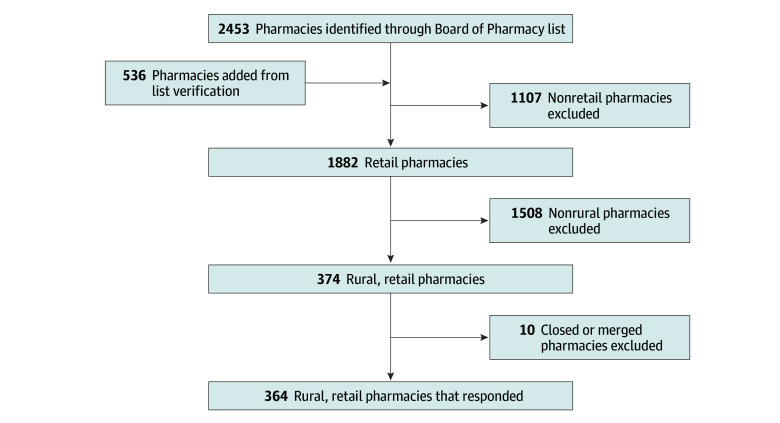
Flowchart Outlining Sampling Frame

If pharmacists reported having the medication in stock, they were asked whether a prescription was necessary. Of the 259 pharmacists asked, 122 (47.1%; 95% CI, 40.9%-53.4%) gave the correct information by saying that a prescription was not necessary, 123 (47.5%; 95% CI, 41.3%-53.8%) said a prescription was required, 8 said they were unsure (3.1%; 95% CI, 1.3%-6.0%), and 6 (2.3%; 95% CI, 0.9%-5.0%) said that a prescription was needed sometimes. The mean (SD) cash price quote for the medication (with no discounts or insurance applied) was $131.04 ($35.83), with a range of $35.98 to $300.

## Discussion

Although the legislative barrier to obtaining naloxone has been addressed in Georgia, naloxone accessibility and availability, especially in rural areas, are still limited. These findings suggest that rural communities face additional barriers related to misinformation and varying costs. Despite these barriers, pharmacists can provide their patients with high-quality care, even if they cannot directly provide naloxone. Some pharmacists identified alternative pharmacies and cost-saving options.

This study did not ask pharmacists why the medication was not stocked in their pharmacies, nor did it examine county-level predictors of naloxone access. Future work should compare the accessibility of naloxone against the rates of opioid overdoses in the county. Despite the existence of a standing order in Georgia, this study shows the necessity for further education and training to ensure the translation and execution of legislative mandates necessary to address this public health crisis.
